# Vitamin and Mineral Deficiencies Are Highly Prevalent in Newly Diagnosed Celiac Disease Patients

**DOI:** 10.3390/nu5103975

**Published:** 2013-09-30

**Authors:** Nicolette J. Wierdsma, Marian A. E. van Bokhorst-de van der Schueren, Marijke Berkenpas, Chris J. J. Mulder, Ad A. van Bodegraven

**Affiliations:** 1 Department of Nutrition and Dietetics, VU University Medical Centre, P.O. Box 7057, Amsterdam 1007 MB, The Netherlands; E-Mails: m.vanbokhorst@vumc.nl (M.A.E.B.S.); m.berkenpas@vumc.nl (M.B.); 2 Department of Gastroenterology, Celiac Centre Amsterdam, VU University Medical Centre, Amsterdam 1007 MB, The Netherlands; E-Mails: cjmulder@vumc.nl (C.J.J.M.); v.bodegraven@vumc.nl (A.A.B.)

**Keywords:** vitamins, minerals, celiac disease, deficiency, adult, Body Mass Index

## Abstract

Malabsorption, weight loss and vitamin/mineral-deficiencies characterize classical celiac disease (CD). This study aimed to assess the nutritional and vitamin/mineral status of current “early diagnosed” untreated adult CD-patients in the Netherlands. Newly diagnosed adult CD-patients were included (*n* = 80, 42.8 ± 15.1 years) and a comparable sample of 24 healthy Dutch subjects was added to compare vitamin concentrations. Nutritional status and serum concentrations of folic acid, vitamin A, B_6_, B_12_, and (25-hydroxy) D, zinc, haemoglobin (Hb) and ferritin were determined (before prescribing gluten free diet). Almost all CD-patients (87%) had at least one value below the lower limit of reference. Specifically, for vitamin A, 7.5% of patients showed deficient levels, for vitamin B_6_ 14.5%, folic acid 20%, and vitamin B_12_ 19%. Likewise, zinc deficiency was observed in 67% of the CD-patients, 46% had decreased iron storage, and 32% had anaemia. Overall, 17% were malnourished (>10% undesired weight loss), 22% of the women were underweight (Body Mass Index (BMI) < 18.5), and 29% of the patients were overweight (BMI > 25). Vitamin deficiencies were barely seen in healthy controls, with the exception of vitamin B_12_. Vitamin/mineral deficiencies were counter-intuitively not associated with a (higher) grade of histological intestinal damage or (impaired) nutritional status. In conclusion, vitamin/mineral deficiencies are still common in newly “early diagnosed” CD-patients, even though the prevalence of obesity at initial diagnosis is rising. Extensive nutritional assessments seem warranted to guide nutritional advices and follow-up in CD treatment.

## 1. Introduction

Celiac disease (CD) is the most common food intolerance in the Western population, and currently represents a major health care issue. The prevalence of CD has been estimate to be 0.5%–1% in different parts of the world [1]. CD is an inflammatory, immune-mediated chronic disease of the mucosa of the proximal small intestine due to irreversible gluten intolerance in genetically susceptible individuals. Gluten refers to a set of amino acid sequences found in the prolamine fraction of wheat, barley and rye. The characteristic histopathological finding is a varying degree of villous atrophy and crypt hyperplasia, primarily in the duodenum and jejunum, with inflammatory changes leading to malabsorption. The first-line, and up-till-now only, treatment is a lifelong strict adherence to a gluten free diet (GFD). All other treatment modalities suppress the intestinal inflammatory response and do not treat the intolerance [2,3].

CD is a multi-system disorder which leads to striking differences in its clinical presentation. When present, gastrointestinal symptoms, including clinically evident malabsorption, may facilitate the diagnosis of CD. Over the last few decades, there appeared to be a changing clinical presentation of CD from the classical malabsorptive picture (diarrhoea, malabsorption and weight loss) towards one of a non-classical presentation with milder, non-specific symptoms such as tiredness, hematologic abnormalities, constipation and/or abdominal distension [4,5,6]. Nowadays, many patients present with no or only minor extra-intestinal symptoms. Indeed, microcytic or macrocytic anaemia, or folate deficiency may occasionally be the only clinical symptom to suggest CD. This leads to a great extent of underdiagnoses in several countries [7]. Currently, 20%–40% of newly diagnosed CD-patients are even classified as overweight (Body Mass Index (BMI) > 25 kg/m^2^) instead of the anticipated underweight [8,9,10,11,12], although the prevalence of obesity seems lower than in the general population [13]. This makes the diagnosis of CD challenging. Greater clinical awareness—especially improved serological testing since the late 1990s, including that for anti-tissue transglutaminase (tTG) antibodies [14,15]—and the appearance of specialized centres has led to earlier recognition of CD-patients.

In the classically presenting CD-patients, malabsorption is frequently encountered [16], and micronutrient deficiencies may arise. Indeed, several studies demonstrate these deficiencies with varying results [17,18,19,20,21].

Our group recently demonstrated the specific functional insufficiency of the proximal small bowel in CD-patients by means of the citrulline generation test, in which the citrulline peak after glutamine administration and its conversion into citrulline in the enterocyte was delayed due to a decreased functional intestinal mass as a consequence of inflammatory changes [22].

Deficiencies of water-soluble vitamins, like B-vitamins, would be expected since they are absorbed in the proximal small bowel, which is the most prominent site affected in CD-patients. However, available data, in particular regarding vitamin B_2_ and B_6_ deficiencies, do not support this in untreated CD-patients [17,23]. Table 1 shows an overview of older and more recent literature on vitamin and mineral deficiencies in adult CD-patients.

**Table 1:** Literature overview on vitamin and mineral status in newly diagnosed adult celiac disease (CD)-patients.

Author, year	Patient Group	Outcome
***Till 2005***		
Hallert,1981 [24]	CD (Folate)	Decreased serum folate concentration abnormality in adult CD. Predictive value of low folate for advice jejunal biopsy.
Stene-Larsen, 1988 [25]	CD (*n* = 3)	Vitamin B_12_ malabsorption by CD is emphasized as a pathogenic mechanism of megaloblastic anaemia.
Crofton, 1990 [20]	Untreated CD (*n* = 8) and healthy controls (*n* = 5) (Zinc)	Impaired turnover and loss of endogenous zinc in mild untreated CD. Zinc levels normal.
Kemppainen, 1995 [26]	Untreated CD (*n* = 40), CD in remission (*n* = 52)(Nutritional status)	Nutritional status quite good in both groups. 15%–38% deficiencies (HB, ferritin, iron or B_12_) in untreated CD compared to 0%–20% in remission CD.
Kemppainen, 1998 [27]	Untreated CD (*n* = 40) (Nutritional status)	Anthropometric and biochemical nutritional status acceptable. Low ferritin and folate (enterocyte) levels, but normalised after 1 year GFD.
Alwitry, 2000 [23]	Celiac disease (Vitamin A)	Case report in vitamin A deficiency and eye deviation
Dahele, 2001 [18]	Untreated CD (*n* = 39) (Vitamin B12)	41% B12 deficient (<220 ng/L), 41% anaemic, and 31% folate deficient f the B_12_ deficient CD-patients.
Dickey, 2002 [28]	CD (*n* = 159) (Vitamin B12)	Low serum B_12_ is common in CD (12%) and is not due to autoimmune gastritis. 10% of B_12_ deficient group had atrophic gastritis. Advice to know B_12_ level before folate supplementation.
Hozyasz, 2003 [29]	Untreated CD Polish (*n* = 18), remission on GFD (*n* = 12) (Vitamin E)	All untreated CD-patients had reduced vitamin E levels. Vitamin A comparable to treated CD.
***2005–2013***		
Harper, 2007 [30]	Untreated (3 mo after diagnosis) CD (*n* = 405) (Anaemia)	Iron deficiency in 31% of male and 19% of females, folate in 12%, B_12_ 5% and anaemia in 20% of CD-patients. Anaemia can not only be explained by nutritional deficiencies.
Dickey, 2008 [17]	Untreated CD (*n* = 35), persistent villous atrophy (*n* = 34, recovered (*n* = 41) (B-vitamins)	No compromised B_2_ and B_6_ in 3 groups.Homocysteine concentrations are inversely associated with serum and red cell folate and with B_12_.
Henri-Bhargava, 2008 [19]	CD (Vitamin E, copper)	Neurological impairment due to vitamin E and copper deficiencies in CD.
Bergamaschi, 2008 [21]	Untreated CD (*n* = 150), after 1 year GFD (*n* = 53) (Anaemia)	34% anaemia at diagnosis. Iron, vitamin deficiencies and anaemia of chronic disease are common in CD. GFD treatment improves anaemia.
Lerner, 2012 [31]	CD (Spanish) (*n* = 22) and CD children (*n* = 120) (Spanish, Israeli), (Vitamin D)	Vitamin D levels correlate negatively with age. 55% of Adult CD-patients had vitamin D deficiency (25-hydroxy < 20 ng/mL) and should be supplemented.

It has been suggested that the current “early diagnosis” of CD might be associated with less vitamin and mineral deficiencies at the moment of diagnosis than the classical CD. Mineral and trace-element status of untreated CD-patients has not been widely studied. There is even a lack of recent reports in the literature (see Table 1) indicating which deficiencies should be checked in newly diagnosed celiacs in Western Europe. Therefore, we aimed to measure essential serum nutritional variables in order to assess the prevalence of vitamin and mineral deficiencies in untreated adult CD-patients from a tertiary referral Celiac Disease Centre, consuming a (gluten containing) standard Dutch (Western) diet before diagnosis. Secondly, we studied the nutritional status and differences in prevalence of vitamin and mineral deficiencies between patients with different grades of intestinal histological damage, nutritional status, and sex.

## 2. Materials and Methods

### 2.1. Patients

Eighty consecutively diagnosed adult patients (aged 18–75 years) with newly diagnosed CD were recruited from the Outpatient Clinic of the VU University Medical Centre, Amsterdam, the Netherlands, during the period 2005–2012. All patients consumed a normal (gluten-containing) Dutch-Western diet until inclusion. The mean daily gluten intake in the Netherlands is around 13 g [32]. Duodenal biopsy specimens were harvested to determine the grade of histological damage due to gluten sensitive enteropathy as classified by Marsh [33] and modified by Rostami [34,35]. Gastric (corpus) biopsies were routinely harvested to determine whether atrophic gastritis was present. CD associated antibodies, *i.e.*, anti-endomysial antibodies (EMA) and anti-tTG antibodies, were determined [36,37]. In addition, HLA-genotyping was performed, to analyse the presence of DQ_2_ and DQ_8_ (heterozygote or homozygote) as a prerequisite for a definitive diagnosis [38]. The diagnosis of CD was based on these histopathological, serological and genetic criteria.

Histopathological classification according to Marsh was used: intraepithelial lymphocytosis, crypt hyperplasia and villous atrophy Marsh IIIA, B and C (respectively, partial, subtotal and total villous atrophy) with or without elevated antibodies. Also, a group was added with low grade histopathological abnormalities Marsh I or Marsh II (lymphocytic enteritis with crypt hyperplasia) with gluten-dependent disorders, in case of elevated antibodies and the HLA-DQ_2_ or HLA-DQ_8_ genotype.

Blood samples were collected as part of routine clinical care. Patients were excluded if they had an established or suspected gastrointestinal abnormality other than CD, such as inflammatory bowel disease (IBD). A representative sample of 24 healthy Dutch subjects (comparable for sex, age and BMI) was added to compare concentrations of vitamin A, B_6_, folic acid and B_12_ (see control subjects characteristics [39]). Both groups were studied in the same period, were living in a similar environment, and measurements were performed at the same clinical chemistry laboratory.

The study protocol was approved by the Medical Ethics Committee (2005, project code 05.153) of the VU University Medical Centre Amsterdam, The Netherlands.

### 2.2. Nutritional Status

Patient characteristics and demographic data (including age (year), body height (m), body weight (kg) and self-reported involuntary weight loss in the past 1 and 6 months) were collected, BMI was calculated and biochemical analyses were performed following diagnosis and before any dietary advice to initiate a GFD. Patients were subsequently classified as “malnourished” when they unintentionally lost more than 10% of their bodyweight in the past 6 months or more than 5% in the past month prior to diagnosis or as having “risk of malnutrition” when 5%–10% of the bodyweight was unintentionally lost in the 6 months before diagnosis. Moreover, patients were classified into 3 groups on the basis of baseline BMI; less than 18.5 kg/m^2^ (underweight), 18.5–25.0 kg/m^2^ (normal weight) and more than 25 kg/m^2^ (overweight, or even “obese” in case of BMI above 30 kg/m^2^) (according to the definition of the World Health Organisation (2000)).

### 2.3. Biochemical Analysis

Fasting venous blood samples were drawn and subsequently analysed at the endocrine and clinical chemistry laboratories of the VU University Medical Centre, Amsterdam. Samples for serum folic acid and serum vitamin B_12_ were analysed by competitive immunoassay (Luminescence, Abbott, IL, USA). Serum vitamin B_6_ and vitamin A status were determined by high-performance liquid chromatography (HPLC). Vitamin (25-hydroxy) D was assessed with a competitive binding protein assay (Diasorin, Stillwater, MN, USA). Zinc status was assessed using Flame Atomic Absorption Spectroscopy (FAAS), serum haemoglobin by colorimetric methods (Cell Dyn Sapphire, Abbott, IL, USA) and ferritin values by electro-chemiluminescence immunoassay “ECLIA” (Roche, Mannheim, Germany) or “ACS CENTAUR” (Bayer, Mijdrecht, The Netherlands).

Vitamin B_6_ and folic acid (both proximally absorbed) and vitamin B_12_ (distally absorbed) were considered to represent the water-soluble vitamin status. Vitamin A and vitamin (25-hydroxy) D were considered to represent the fat-soluble vitamin status, although sun exposure, even in The Netherlands, may have a strong influence on serum levels of vitamin D. Haemoglobin and ferritin levels below the reference ranges listed were used to establish a respective diagnosis of anaemia, iron-deficiency or iron-deficiency anaemia when both haemoglobin and ferritin were below reference ranges. Patients with a serum value below the lower limit of the reference value were considered “deficient”. Reference values for the different parameters are displayed in Table 3.

### 2.4. Statistical Analysis

Data were tested for normal distribution and presented as means ± SD. The percentage of patients with values below the reference value and absolute number of deficient patients were additionally calculated for all assessed serum vitamin and mineral concentrations.

Data were analysed for the total group, and in stratified subgroups by sex, histological damage (Marsh classification) and BMI. To determine differences with regard to gender, a Student’s *t*-test was applied in case of continuous data and Pearson’s Chi-Square tests (χ²) in the case of comparing proportions (% of deficient patients). Analysis of variance (ANOVA), with a Bonferroni correction when a statistical significant difference was achieved, was used to compare more than two groups. A Mann-Whitney U test (Wilcoxon) or in case of more than 2 variables, a Kruskal-Wallis test was applied for variables not found in a normal distribution. The level of statistical significance was determined a priori at *p* < 0.05. Statistical analyses were performed using SPSS (Statistical Package for Social Sciences Inc., Chicago, IL, USA—Windows version 20.0).

## 3. Results

### 3.1. Patient Characteristics and Nutritional Status

Patient characteristics are shown in Table 2; two-thirds of the population was female. Male patients were significantly older than female patients (*p* = 0.006). Approximately 46% (37/80) of the patients had partial villous atrophy (Marsh IIIA) and 42.5% showed subtotal (20/80) or total villous atrophy (14/80) (Marsh IIIB or IIIC, respectively). Histologically and serologically atrophic (corpus) gastritis was ruled out by a pathologist experienced in intestinal histology. Some of the CD patients reported to have used vitamin and mineral supplements before diagnosis of CD was made: 18 (22.5%) a multivitamin, folic acid or vitamin B-complex, 7 (8.8%) iron supplements and 14 (17.5%) a calcium supplement. The anthropometric data of 24 healthy controls were comparable to those of the patients included.

Patients had, on average, lost 2.4% (±6.3%) of their bodyweight during the 6 months prior to diagnosis. Approximately 17% was classified as malnourished (>10% weight loss) and 5% as being at risk of malnutrition (5%–10% previous weight loss). Six out of 80 patients (7.5%), and only females, were classified as underweight, whereas 29% (23/80) of patients were classified as overweight (female:male ratio =1:1). Of the overweight patients, 26% were even obese (6/80 of the total group, with female:male ratio = 1:1).

**Table 2:** Patient characteristics of untreated adult CD-patients by gender and compared to healthy controls.

CD Patients					Healthy Controls *
*N*		80			24
Sex		All	Female (52)	Male (28)	F14/M11
Age (year)	mean ± SD (range)	42.8 ± 15.1 (18–75)	39.5 ± 14.3 ^	49.1 ± 14.9	43.0 ± 12.9
Height (m)	mean ± SD	1.73 ± 0.1	1.68 ± 0.09 ^	1.80 ± 0.08	1.76 ± 0.07 *^a^
Weight (kg)	mean ± SD	70.6 ± 15.3	66.0 ± 14.3 ^	79.1 ± 13.8	75.5 ± 11.6
BMI (kg/m^2^)	mean ± SD	23.6 ± 4.0	23.2 ± 4.2	24.3 ± 3.6	24.1 ± 2.6
Marsh classification *N* (%)	<18.5	6 (7.5%)	6 (22.5%)	0	0
	18.5–25	51 (63.8%)	31 (59.6%)	20 (71.4%)	18 (72%)
	>25	23 (28.8%)	15 (28.8%)	8 (28.6%)	27 (28%)
	I/II #	9 (11.3%)	6 (11.5%)	3 (10.7%)	
	IIIA	37 (46%)	23 (44.2%)	14 (50.0%)	
	IIIB	20 (25%)	10 (19.2%)	10 (35.7%)	
	IIIC	14 (17.5%)	13 (25%)	1 (3.6%)	
Antibodies *N* (%)	Negative	17 (21.3%)	11 (21.1%)	6 (22.0%)	
EMA	doubtful	1 (1.3%)	1 (1.9%)	0	
	weak positive	5 (6.3%)	5 (9.6%)	0	
	positive	14 (17.5%)	10 (19.2%)	4 (14.3%)	
	strong positive	38 (47.5%)	23 (44.2%)	15 (53.6%)	
	n.d.	5 (6.3%)	2 (3.8%)	1 (3.6%)	
tTG	Negative	16 (20%)	9 (17.3%)	7 (25%)	
	doubtful	4 (5%)	3 (5.8%)	1 (3.6%)	
	weak positive	10 (12.5%)	9 (17.3%)	1 (3.6%)	
	positive	18 (22.5%)	12 (23.1%)	6 (21.4%)	
	strong positive	31 (38.8%)	19 (36.5%	12 (42.9%)	
	n.d.	1 (1.3%)	0	1 (3.6%)	
tTG (U/mL)	mean ± SD (range)	171 ± 402 (3.2–2500)	124 ± 378 (4–2500)	274 ± 445 (3.2–1999)	
CD genotypes	DQ_2(hetero-/homozygote)_	62 (77.5%)_(56/6)_	42 (80.8%)_(37/5)_	20 (71.4%)_(19/1)_	
	DQ_8(hetero-/homozygote)_	5 (6.3%)_(3/2)_	3 (5.8%)_(2/1)_	2 (7.1%)_(1/1)_	
	DQ_2_ and DQ_8_	3 (3.8%)	2 (3.8%)	1 (3.6%)	
	DQ_2_ nor DQ_8_	3 (3.8%)	1 (1.9%)	2 (7.1%)	
	n.d.	7 (8.8%)	4 (7.7%)	3 (14.3%)	

BMI: Body Mass Index, EMA: anti-endomysial antibodies, tTG: anti-tissue transglutaminase, ^ Significantly different from men (*p* < 0.05) by Student’s *t*-test, n.d. (not determined), # low grade histopathological abnormalities with HLA-DQ_2_ and/or DQ_8_ and elevated antibodies (EMA and/or tTG), * variables NS (not statistically significant from CD-patients (*p* < 0.05) by Mann-Whitney U test), *^a^ statistical trend *p* = 0.05.

**Table 3:** Serum concentrations of nutritional biochemical parameters of untreated adult CD-patients (compared to healthy controls).

Serum Vitamin/Mineral [Reference Value]	Serum Concentration ^				Percentage deficient patients #			
	All	Male	Female	Healthy Controls	All	Male	Female	Healthy Controls
Vitamin A [1.2–3.0 nmol/L]	2.0 ± 0.7 0.1–4.0 (53)	2.2 ± 0.9 (17)	1.9 ± 0.6 (36)	2.5 ± 0.6 (1.4–4.0) ^%a^ (25)	7.5% (4/53)	11.8% (2/17)	5.6% (2/36)	0% (0/24)
Vitamin B_6_ [13–80 nmol/L]	92.2 ± 142.7 6.0–593.0 (62)	56.1 ± 87.1 (20)	109.4 ± 160.8 (42)	70.1 ± 74.5 (21–384) (25)	14.5% (9/62)	25.0% (5/20)	9.5% (4/42)	0% (0/24) ^$a^
Folic acid [>5.6 nmol/L]	15.1 ± 15.0 2.1–90.1 (80)	10.4 ± 6.6 (28)	17.6 ± 17.6 *^a^ (52)	20.4 ± 18.1 (4.4–91) ^%b^ (25)	20.0% (16/80)	28.5% (8/28)	15.4% (8/52)	4.2% (1/14) ^$b^
Vitamin B_12_ [150–700 pmol/L)	231.2 ± 104.3 64.0–590.0 (80)	222.6 ± 75.6 (28)	235.7 ± 117.4 (52)	272.6 ± 112.5 (100–453) (25)	19.0% (15/80)	22.2% (6/27)	17.3% (9/52)	16.6% (4/24)
Zinc [11–19 nmol/L]	10.3 ± 2.1 6.3–14.8 (40)	10.4 ± 2.2 (16)	10.2 ± 2.0 (24)		66.7% (26/40)	62.5% (10/16)	70.8% (17/24)	
Vitamin (25-hydroxy) D [30–150 nmol/L]	64.8 ± 26.8 29–120 (21)	64.5 ± 35.3 (8)	65.0 ± 21.6 (13)		4.8% (1/21)	12.5% (1/8)	0 (0/13)	
Haemoglobin [M 8.5–11/F 7.5–10 mmol/L]	8.0 ± 1.1 5.0–10.2 (71)	8.9 ± 0.8 (25)	7.5 ± 0.9 *^b^ (46)		32.4% (23/71)	24.0% (6/25)	37.0% (17/46)	
Ferritin [20–250 µG/L]	48.1 ± 83.9 3.0–451.0 (39)	110.9 ± 147.2 (10)	26.4 ± 26.2 (29)		46.2% (18/39)	30.0% (3/10)	51.7% (15/29)	

^ Successively expressed as: mean ± SD, range, (*N*), * statistically significantly different from men ( Student’s *t*-test), *^a^*p* = 0.04, *^b^*p* < 0.001, # NS (not statistically significant (*p* < 0.05) by Pearson’s Chi-Square tests (χ²)), ^%^ statistically significantly different from CD by Mann-Whitney U test, ^%a^*p* = 0.006, ^%b^*p* = 0.030, ^$^ statistically significant by Pearson’s Chi-Square tests (χ²), ^$a^*p* = 0.049, ^$b^*p* = 0.066.

### 3.2. Biochemical Analyses

Serum concentrations of vitamins and minerals of the untreated CD-patients are shown in Table 3. CD-patients were most frequently deficient for folic acid (20%, 16/80), followed by vitamin B_12_ (19%, 15/79), vitamin B_6_ (14.5%, 9/62), vitamin A (7.5%, 4/53) and vitamin (25-hydroxy) D (4.5%, 1/21), respectively. Approximately 67% (26/39) of the patients had zinc deficiency, 32.4% (23/71) had anaemia, 46.2% (18/39) had insufficient iron storage evidenced by low ferritin and 25% (8/40) had iron-deficiency anaemia. CD-patients had lower values of vitamin A and folic acid than healthy controls. Overall, vitamin deficiencies were barely seen in healthy controls, with the exception of vitamin B_12_. None of the healthy controls showed deficient levels (below the reference values) for vitamin A and vitamin B_6_ and only one for folic acid.

Ten patients (12.5%) were not deficient for any of the assessed vitamins and minerals. These patients had similar base-line characteristics to the rest of the CD group. The remaining 70 patients (87.5%) were deficient in at least one of the nutritional parameters and 43 (53.8%) for two or more parameters.

Table 3 depicts the proportion of deficient patients, stratified by sex. As anticipated, serum haemoglobin concentrations were lower in women than in men (*p* < 0.001). While, on the other hand, serum folic acid concentrations were lower (*p* = 0.040) in men when compared to women. No statistically significant difference was found for any of the other nutritional parameters when comparing males and females. Notwithstanding, multivitamin use (including folic acid or vitamin B supplement) at their own volition or prescribed by the GP was more prevalent in women than men (30% *vs.* 13%). A statistical trend was seen for vitamin B_6_, suggesting that men might be more often deficient than women (*p* = 0.097).

### 3.3. Association between Vitamin and Mineral Concentrations and Histological Damage (Marsh-Classification)

Table 4 depicts the mean serum vitamin and mineral values by Marsh strata. No statistically significant differences were found between the serum vitamin and mineral concentrations across the four Marsh strata, except for ferritin. Serum ferritin values decreased when the villous atrophy score increased (*p* = 0.041).

**Table 4:** Mean serum concentrations of vitamins and minerals (±SD) in untreated CD-patients by Marsh stratum.

Serum Vitamin/Mineral	Marsh Stratum			
	I/II # (*n* = 9)	IIIA (*n* = 37)	IIIB (*n* = 20)	IIIC (*n* = 14)
Vitamin A (nmol/L)	2.3 ± 0.4 (4/9)	2.0 ± 0.8 (28/37)	1.9 ± 0.44 (11/20)	1.9 ± 0.8 (10/14)
Vitamin B_6_ (nmol/L)	129.8 ± 115.0 (7/9)	107.6 ± 170.7 (29/37)	55.7 ± 43.5 (15/20)	86.8 ± 163.9 (11/14)
Folic acid (nmol/L)	14.1 ± 8.5 (9/9)	14.7 ± 12.9 (37/37)	13.9 ± 15.5 (20/20)	18.4 ± 22.4 (14/14)
Vitamin B_12_ (pmol/L)	282.4 ± 151.7 (9/9)	225.2 ± 91.1 (37/37)	216.5 ± 80.2 (20/20)	234.7 ± 131.8 (14/14)
Vitamin (25-hydroxy) D (nmol/L)	89.3 ± 27.0 (3/9)	52.7 ± 16.4 (7/37)	63.0 ± 31.4 (6/20)	69.2 ± 29.2 (5/14)
Zinc (nmol/L)	11.0 ± 1.7 (5/9)	10.8 ± 2.3 (19/37)	9.8 ± 1.7 (11/20)	8.8 ± 1.7 (5/14)
Haemoglobin (mmol/L) *	8.5 ± 0.5 (7/9)	8.0 ± 1.2 (33/37)	8.0 ± 1.1 (18/20)	7.9 ± 1.0 (13/14)
Ferritin (µG/L) ^	123.8 ± 167.3 (6/9)	47.6 ± 66.3 (18/37)	18.8 ± 17.6 (10/20)	17.2 ± 22.0 (5/14)

# Low grade histopathological abnormalities with HLA-DQ_2_ and/or DQ_8_ and elevated antibodies (EMA and/or tTG), * gender specific Females I/II 8.2 ± 0.29; IIIA 7.4 ± 0.95; IIIB 7.3 ± 0.84; IIIC 7.8 ± 0.97 and Male I/II 8.9 ± 0.78; IIIA 8.9 ± 0.96; IIIB 9.0 ± 0.67; IIIC 8.8 ± 0.72, ^ statistically significantly different (*p* = 0.041) by Kruskal-Wallis test.

### 3.4. Association between Vitamin and Mineral Concentrations and Nutritional Status

Table 5 depicts the mean serum concentrations of vitamins and minerals per BMI stratum. A trend was observed for underweight patients having a slightly higher serum folic acid concentration than patients with normal weight or overweight patients (*p* = 0.058). Besides, patients with >10% unintentional weight loss in the past 6 months had higher vitamin A and (a trend for) higher vitamin B_6_ (*p* = 0.09) levels than patients without weight loss. Otherwise, no differences were observed between the different classes of nutritional status.

**Table 5:** Mean serum concentrations of vitamins and minerals (±SD) in untreated CD-patients by nutritional status (BMI stratum and unintentional weight loss).

Serum Vitamin/Mineral	BMI Stratum (kg/m^2^) ^				Weight Loss (% in past 6 months)	
	Under-Weight (<18.5) (*n* = 6)	Normal Weight (18.5–25.0) (*n* = 51)	Over-Weight (>25) (*n* = 23)		Well-Nourished (0%–10%) (*n* = 64)	Mal-Nourished (>10%) (*n* = 13)
Vitamin A (nmol/L)	1.7 ± 0.7 (5)	2.0 ± 0.7 (35)	2.0 ± 0.6 (13)		1.8 ± 0.6 (40)	2.6 ± 0.7 # (11)
Vitamin B_6_ (nmol/L)	128.5 ± 207.8 (6)	99.3 ± 146.2 (39)	63.1 ± 109.0 (17)		68.1 ± 102.0 (48)	206 ± 240.8 (11)
Folic acid (nmol/L)	33.0 ± 29.9 (6)	14.5 ± 13.4 (51)	11.8 ± 10.0 (23)		13.4 ± 11.2 (64)	23.5 ± 27.1 (13)
Vitamin B_12_ (pmol/L)	216.3 ± 57.3 (6)	226.2 ± 114.5 (51)	246.0 ± 90.4 (23)		227.6 ± 104.0 (64)	250.8 ± 117.7 (13)
Vitamin (25-hydroxy) D (nmol/L)	52.0(1)	63.4 ± 28.0 (11)	68.0 ± 27.9 (9)		67.1 ± 27.1 (17)	55.0 ± 26.5 (4)
Zinc (nmol/L)	8.8 ± 0.3 (2)	10.3 ± 2.1 (26)	10.6 ± 2.2 (12)		10.5 ± 2.1 (31)	9.8 ± 2.1 (7)
Haemoglobin (mmol/L)	7.6 ± 0.7 (6)	8.0 ± 1.1 (45)	8.2 ± 1.2 (20)		8.1 ± 1.1 (56)	7.8 ± 1.4 (12)
Ferritin (µG/L)	25.0 ± 2.1 (3)	34.9 ± 38.4 (26)	89.0 ± 151.9 (10)		48.5 ± 90.4 (33)	46.2 ± 38.3 (5)

^ NS (not statistically significant (*p* < 0.05) by Kruskal-Wallis test, # statistically significantly different from well-nourished patients (Student *t*-test), *p* = 0.001.

## 4. Discussion

The present study showed that the majority of an “early diagnosis” adult untreated CD patient group (with so-called non-classical presentation) in the Netherlands, had at least one, and often several, serum vitamin or mineral deficiencies at diagnosis. Almost 90% of CD-patients were found to be deficient in at least one or more of the assessed nutritional parameters, and half of patients were deficient for two or more nutritional serum variables. This was observed to be unrelated to severity of clinical presentation, nutritional status or (semi-quantified) histopathological damage (score).

In this study, no statistically significant difference in serum vitamin and mineral concentration was found between men and women, although (multi)vitamin use prior to diagnosis was more prevalent in women than men (30% *vs.* 13%). Information is lacking whether they took the vitamins at their own volition, or if they were prescribed by the general practitioner. In addition, the assessed serum deficiencies were independent of Marsh stratum (Table 4), nutritional status (Table 5) and age (data not shown). Folic acid deficiency was observed in 20% of the untreated CD-patients in this study. The prevalence of folate deficiency varies from 18% to 90% in varying older and newer reports of CD-patients [17,18,24,26]. In studies from Scotland and Finland, folate deficiency was reported in 42% [18] and 37% [27] of the untreated CD-patients, respectively. The difference in prevalence might at least partially be explained by technical aspects of measurement of “folate” (which is the natural form of folic acid) and “folic acid” since bioavailability of folic acid is twice that of folate [40]. Macrocytic anaemia in untreated CD-patients is usually caused by folate deficiency. In two large European studies, anaemia, mostly attributed to malabsorption, was reported to be present in 20%–34% of untreated CD-patients [20,30]. This corresponds with our results in which 25% of the CD-patients suffered from iron-deficiency anaemia.

The presence of vitamin B_6_ deficiency has been reported in two studies, albeit in children with “acute celiac disease”. A decreased pyridoxal phosphate was reported in serum samples and in duodenal mucosa, suggestive of vitamin B_6_ deficiency [41,42] and indicative for decreased levels of vitamin B_6_ in untreated CD children. We found water-soluble vitamin deficiencies (B_6_, folic acid and B_12_) in approximately one in seven (B_6_) to one in five (folic acid and B_12_) untreated CD-patients. This was despite the fact that more than 20% of the patients were using a prescribed or over-the-counter multivitamin/vitamin B-complex or a folic acid supplement before diagnosis.

Vitamin B_12_ deficiency was frequently observed in our CD-patient group (19%), in accordance with earlier studies, notably also in those without atrophic gastritis [17,18,26,27,28]. Intriguingly, this vitamin is typically absorbed in the terminal ileum. Apparently, the distal small bowel is functionally more affected than previously believed, based on patho-histological analysis of distal small bowel biopsy samples [43]. Vitamin B_12_ deficiency in untreated CD-patients has been confirmed in several previously conducted European studies [18,25,26,27,28], ranging from 12% up to 41%. It may be hypothesized that vitamin B_12_ deficiency is a result of a dysfunctional intrinsic factor [44]. Dickey reported that low vitamin B_12_ concentrations in CD were not due to auto-immune gastritis [28]. In this study, none of the patients had histologically or serologically demonstrated atrophic gastritis and, therefore, this was unlikely to be responsible for vitamin B_12_ deficiencies in CD.

In various small studies in Europeans, deficiencies of the fat soluble vitamins A [23,27], E [19,29] and D [45] in untreated CD-patients have been previously reported. The latter has been associated with osteomalacia. In clinical practice, many believe that the clinically relevant lower limit for vitamin D deficiency should be increased. As a consequence, the displayed (relatively low) deficiency percentages can therefore be an underestimation. The observed 7.5% of vitamin A deficient patients is less than the percentage reported in Finland (14%) for this vitamin [27]. From healthy subject studies, it is known that the vitamin A body storage is usually stable and sufficient for approximately one to two years [46]. This is confirmed by our healthy sample, in which none of the subjects showed a deficiency.

A majority of our untreated CD-patients had a zinc deficiency, which can probably be explained by increased endogenous losses of zinc, rather than abnormal zinc absorption [20]. Clinical relevance of zinc deficiency remains inconclusive and additional research is warranted. However, it is known that CD is associated with a wide array of skin lesions and manifestations, which may be partly ascribed to zinc deficiency [47,48,49,50], and cell mediated immunity and antioxidant buffer capacity may be compromised due to it as well [51].

Possible explanations for the high prevalence of nutritional deficiencies in untreated CD-patients might be an insufficient nutritional intake. This is supported by the presence of malnutrition in this cohort: 17% was malnourished based on the usual definition of >10% involuntary body weight loss prior to diagnosis and 7.5% had a BMI <18.5 kg/m^2^. However, this seemed unlikely since patients did not report any changes (intentional or involuntary) in their habitual diet before diagnosis. Moreover, the observed presence of high serum folate levels, particularly in those classified as underweight, were contradictory as well. On the other hand, increased faecal losses of nutrients as a result of malabsorption might (partially) explain the high prevalence of deficiencies. However, since most patients did not report any clinical sign of malabsorptive diarrhoea, losses via the stools were thought to be of limited importance as well. Nevertheless, the observed findings in this study, sharing deficiencies in water and fat soluble vitamins, zinc and iron, indicate that maldigestion, malabsorption or a structurally moderately inadequate intake might have been present long before the clinical diagnosis of CD was established. It is known that the delay in diagnosing CD can be more than a decade [52]. One may hypothesize that this might be due to functional changes of the intestinal tract or due to changes in the intestinal microbiome [53]. In this study, even patients with mild CD (low grade pathohistological abnormalities) showed nutritional deficiencies and weight loss. A finding that was recently corroborated in a large Italian cohort showed that mild (histopathological) enteropathy did not necessarily mean mild intestinal dysfunction since, also in this group, alterations in bone marrow density and laboratory parameters were reported [54]. Remarkably, only female patients presented as underweight (approximately one in four females), while the female-male ratio was similar in the overweight and obese patients. A statistical trend for higher serum vitamin A and B concentrations was observed in patients classified as malnourished (low BMI or >10% unintentional weight loss prior to diagnosis). This may be explained by their more so-called classical CD presentation, which may trigger use of supplements due to a greater physicians’ or patients’ awareness when observing clinical signs of malnutrition.

Some considerations arise when interpreting the presented results: not all data were available in all subjects and some subgroups were relatively small, precluding detection of small effects or the drawing of firm conclusions in subgroups. Vitamin or mineral supplement use, whether at the patient’s own initiative or prescribed by the GP, was based on self-reported information and can therefore be underestimated if patients forget to mention these “medicinal supplements”. However, real numbers and frequencies of deficiencies or anaemia might be underestimated, since some patients were already taking these supplements before the initial diagnosis of CD, potentially leading to misclassification as “non-deficient” in this study. Besides, serum values of almost all vitamins or minerals do not fully represent total body stock or physiological function.

Based on our experience and supported by a recently published guideline on CD [3], we suggest monitoring body weight at diagnosis and nutritional serum parameters; at least vitamin B_6_, folic acid, B_12_ and zinc and in any case (25-hydroxy) D of the fat soluble vitamins (due to its connection with presence of osteomalacia). Moreover, we suggest follow-up until serum values are at satisfying levels or upon indication (for instance, if bone density deviations, chronic diarrhoea, or skin lesions are present). Practically, a standard complete multivitamin supplement (100%–300% of RDA) should be considered for every newly diagnosed CD-patient. Continuation time has yet to be determined, since patients are at risk for vitamin deficiencies even after 10 years of a GFD [55]. Evidently, hypervitaminosis should be avoided, in particular regarding pyridoxine and iron [56,57]. It is demonstrated that bone-mineral density and nutritional status can improve after a GFD treatment [58] as well as that of general well-being, which can improve after vitamin B supplementation in CD-patients on a GFD [59].

## 5. Conclusions

In conclusion, deficiencies of vitamins or minerals are frequently observed in untreated adult Dutch CD-patients using a Western diet, although they are currently diagnosed earlier than in the previous century. This was observed even in obese patients. Almost 90% of the newly diagnosed CD-patients had one or more nutritional deficiencies. Malnutrition, expressed as an involuntary weight loss or being underweight, was found in 16% and 7.5% of patients, respectively, while overweight status (BMI > 25 kg/m^2^) was present in almost 30% of the patients. Therefore, these results indicate that extensive nutritional assessment of body weight and serum nutritional parameters should be an integral part of celiac disease treatment to guide nutritional advices and follow-up in CD-treatment by means of an adequately composed individual-based, gluten free diet.

## Financially Support

This study was funded in part by a grant received from the Dutch Society for Gastroenterology (Gastrostart). Gastrostart had no involvement in any of the study tasks.
